# Supplementary material for: Complexome profiling of the Chlamydomonas psb28 mutant reveals TEF5 as an early PSII assembly factor
Source: Plant Cell. 2025 Mar 17;37(6):koaf055. doi: 10.1093/plcell/koaf055 (PMC12164590; doi:10.1093/plcell/koaf055)
Supplement: koaf055_Supplementary_Data [file koaf055_supplementary_data.zip › psb28_interactive.zip!/plots/intensity/qualityPlots/peptides/_Cre01.g037200.t1.2.html]

### Cre01.g037200.t1.2

Trivial name:   
SeqCoverage [median]: 29.10  
Mass: 11.43 kDa  
  
 

| Sequence | MissedCleavage | Mass [Da] | wtIntensity | muIntensity | Score |
| --- | --- | --- | --- | --- | --- |
| AAEKGDVLEGGVAEVGVK | 1 | 1726.91 | 4758.61 | 7894.86 | 126.26 |
| ATDADEKLIEEMKR | 2 | 1647.81 | 26535.78 | 29420.78 | 130.24 |
|  |  |  | 31294.4 | 37315.6 |  |
